# Factors that contribute to the perceived treatment effect of spinal manipulative therapy in a chiropractic teaching clinic: a qualitative study

**DOI:** 10.1186/s12998-024-00554-z

**Published:** 2024-12-18

**Authors:** Patrick Boylan

**Affiliations:** Logan University, 1851 Schoettler Rd, Chesterfield, MO 63139 USA

**Keywords:** Spinal manipulation, Chiropractic, Spine pain, Treatment outcome, Therapeutic alliance, Doctor-patient relationship, Chiropractor-patient relationship, Contextual factors, Education, Qualitative research

## Abstract

**Background:**

Despite the progress made in better understanding the potential mechanisms of spinal manipulative therapy (SMT) and its treatment effects, a knowledge gap continues to exist when identifying the specific factors that contribute to the perceived treatment effect associated with SMT. The purpose of the study was to explore the perceptions of chiropractic clinicians, interns, and patients regarding what factors during a doctor-patient encounter contribute to the perceived treatment effect associated with SMT.

**Methods:**

This study used convenience sampling to enroll participants from a chiropractic teaching clinic in the United States. Semi-structured interviews were used as the main form of data collection, which took place from January-April 2024. The data was subsequently analyzed using thematic analysis and organized into themes through an iterative open coding process.

**Results:**

Six rounds of interviews were conducted for a total of 18 interviews. Each round consisted of one patient who received treatment including SMT, one intern who performed the treatment, and one clinician who oversaw the treatment. After analyzing the interview data, the following five themes were identified: Treatment Outcome, Therapeutic Alliance, Adjunctive Therapies, Significance of Cavitation, and Psychomotor Skills. Each theme consisted of multiple subthemes which were mentioned by the participant groups at varying frequencies. Patients frequently mentioned the importance of improvement in symptoms following treatment, as well as good communication skills and the use of adjunctive therapies. Interns valued functional change following treatment, while clinicians focused on confidence levels and psychomotor skills. There were differing views on the significance of cavitation, ranging from indifference to an indication of a successful treatment.

**Conclusion:**

This qualitative study identified several themes which describe factors that may contribute to the perceived effect associated with SMT. In addition to the psychomotor skills required to perform SMT, educators and practitioners should consider factors such as the therapeutic alliance between patient and provider, use of adjunctive therapies, and assessment of the outcome associated with the intervention.

**Supplementary Information:**

The online version contains supplementary material available at 10.1186/s12998-024-00554-z.

## Background

Spine-related pain is a leading cause of disability and a major socioeconomic burden worldwide [1, 2]. In the United States, low back and neck pain account for more healthcare spending than any other condition [3]. People experiencing back or neck pain often seek care from complementary and alternative providers such as chiropractors [4]. Spinal manipulative therapy (SMT) is a commonly-used treatment technique employed by chiropractors that often involves a high-velocity, low-amplitude (HVLA) thrust [5]. The intended outcome is to restore motion of the affected joints, thereby decreasing pain levels and associated disability [6]. SMT is now often recommended as a viable option for conservative management of spinal pain [7].

While great strides have been made to better understand exactly how SMT decreases pain and improves function [8], the exact underlying mechanism remains unclear [9]. The mechanisms of SMT are likely multifactorial and include some combination of biomechanical, neurophysiological, and contextual components [10]. Biomechanical factors include the amount of force and speed produced by the thrust, resulting in movement of facet joints and changes in muscular reflex [6]. The fact that SMT also results in a neural response, both centrally and peripherally, is now widely accepted [11]. In addition to biomechanical and neurophysiological factors, contextual factors such as the doctor-patient relationship can also have an impact on treatment outcomes associated with SMT [12].

Few studies have explored the perceptions of patients and practitioners regarding what is valued during treatment sessions that include SMT. Maiers et al. [13, 14] used a qualitative approach to explore patients’ perceptions of chiropractic treatment for back and neck pain that included SMT. The researchers concluded that relationship dynamics between doctor and patient could be a key factor in perceiving benefit and satisfaction with treatment. Similarly, Plank et al. [15] used semi-structured interviews to explore patients’ perceptions and expectations of various manual therapy treatments. They found that patients’ expectations of treatments were heavily influenced by their previous experiences and social environments, including interactions with the practitioner administering the treatment. These findings highlight the importance of the doctor-patient relationship, as well as patients’ understanding of their pain and the treatments that aim to provide relief.

Pinpointing the exact underlying mechanisms by which the positive outcomes associated with SMT are produced, and which types of patients will have a favorable response to the treatment has been the focus of recent research. Gorrell et al. [16] attempted to synthesize quantifiable kinetic factors of SMT, such as preload and peak force, rate of force application, and thrust duration. The researchers found that there is wide variability in these characteristics, likely due to individuality of clinicians and differences in measurement techniques. Pasquier et al. [17] sought to identify factors associated with clinical responses following SMT, and found that patient expectations and comfort during the procedure were associated with positive outcomes.

More evidence shows that contextual factors also play a key role in patients’ responses to SMT [18]. These contextual factors include components of the doctor-patient relationship such as developing a strong therapeutic alliance, using effective communication skills, and setting expectations. Recently, recommendations have been made to modernize the teaching of SMT by incorporating communication and context into educational practices [19].

From an educational standpoint, there is wide variation in how SMT is taught within chiropractic institutions [20]. Additionally, there seems to be a weak association between subjective and objective measures of performance when evaluating trainees’ skills related to SMT. Pasquier et al. [21] evaluated the relationship between biomechanical parameters, such as force-sensing tables, paired with direct observation from clinicians and feedback from recipients of SMT. Their findings indicate that there seems to be some discrepancy between subjective assessments of performance and the objective measures that were used.

Despite the progress that has been made to better understand the potential mechanisms of SMT and its treatment effects, a knowledge gap continues to exist when identifying the specific factors that contribute to the effective implementation of SMT. This lack of understanding of the factors that contribute to the perceived treatment effect of SMT could lead to misconceptions regarding its benefits. This could affect teaching practices related to SMT, as well as implementation of the treatment into clinical practice. Qualitative research will illuminate this topic by exploring the perceptions of various stakeholders involved in the utilization and education of SMT, including patients, interns, and clinicians.

The purpose of this study was to explore the perceptions of chiropractic clinicians, interns, and patients regarding what factors during a doctor-patient encounter contribute to the perceived treatment effect associated with SMT. This study sought to answer two research questions. First, what factors from the doctor-patient interaction contribute to the perceived effect of SMT in patients with spine pain during a treatment visit that includes SMT? Secondly, how does the importance of these factors vary among the stakeholders involved, including patients, interns, and clinicians?

## Methods

### Design

This qualitative study explored the perceptions of chiropractic clinicians, interns, and patients regarding what factors during a doctor-patient encounter contribute to the perceived treatment effect associated with SMT. The study was approved by the Logan University Institutional Review Board on January 24, 2024 (Control #RD01242024609). The setting of the study was a chiropractic teaching clinic, where final year chiropractic interns gain clinical experience by seeing patients under the supervision of licensed clinicians. The study involved conducting interviews with patients who received HVLA SMT for spine pain, interns who administered the treatment, and clinicians who oversaw the treatment visit. This was done in an attempt to better understand the experience of SMT from the perspectives of multiple parties who were involved in the treatment session.

### Participants

The study included adult new patients (age 18–55) presenting to a chiropractic teaching clinic with non-specific back or neck pain who were deemed candidates for HVLA SMT after their initial consultation and examination, and who were interested and able to participate in semi-structured interviews. This excluded patients who had been seen at the clinic previously, patients who were students at the university, pregnant patients, and patients whose symptoms were suspected to be attributable to a specific cause and/or presented with a contraindication to receive HVLA SMT (trauma, cancer, infection, etc.). Older adults and pregnant patients were excluded as the treatment consisted of HVLA spinal manipulation specifically, which some clinicians may be less likely to perform in these special populations. Additionally, some of the questions in the interviews pertained to the presence or absence of cavitation, which could potentially be affected by the biomechanical changes associated with aging and pregnancy.

Faculty clinicians and third-year interns who were participating at clinical rotations within the clinic between January 2024 and April 2024 were also invited to participate in semi-structured interviews to explore their perceptions. This included interns who utilized HVLA SMT to treat a patient fitting the eligibility criteria, and a supervising clinician who directly oversaw that treatment.

To enroll participants, this study used a convenience sample, which is enrollment based on availability and accessibility [22]. Patients presenting to the clinic who fit the eligibility criteria were approached after their first visit and asked if they were interested in participating. None who were approached refused to participate or dropped out of the study.

### Semi-structured interviews

Data for this project was collected through semi-structured interviews which are often used in qualitative research to provide a framework for asking participants open-ended questions with the hope of exploring a particular construct [23]. The interview guide can be found in Supplementary File 1. To increase the validity of the interview guide, the framework was shared with researchers with qualitative experience in conducting semi-structured interviews on similar topics. That feedback was implemented prior to piloting the interview guide on participants who matched the inclusion criteria, after which additional feedback was used to make any revisions necessary. After this process of piloting the interview guide through mock interviews and revising the guide as needed, a final version was developed to be used in the study.

The interviewer had no established relationship with the patient participants included in the study. However, familiarity existed between the researcher and clinician/intern participants, as he served as a faculty clinician at the institution during the time of the study. Participants were informed that the researcher was a faculty member at the institution and was conducting a study to learn more about the effects of SMT.

The interviews took place in a private office within the clinic and lasted between 10 and 20 min. Only the researcher and participant were present during the interview. The audio was recorded and transcribed using Zoom software with participants’ informed consent [24]. Field notes were also taken by the researcher during the interviews. No repeat interviews were conducted, and transcripts were not provided for participants.

Interviews were conducted by the author of the study, who was a male Doctor of Chiropractic and EdD candidate during the time of the study. The project was completed as part of the research requirement within a Doctor of Education in Health Professions program. The interviewer had received training in qualitative research methods during the coursework in the program.

### Data analysis

The analysis of qualitative data was completed using the thematic analysis method described by Braun and Clarke [25]. This analytical method is a six-step, iterative process that involves becoming familiar with the data, generating codes, generating themes, reviewing themes, defining and naming themes, and producing the report. This process was conducted and repeated after each round of interviews until the point of data saturation was reached, at which no new codes or themes were generated [26]. Dedoose software was used to help organize and convert the data into themes [27]. After each round, transcripts were edited and reviewed for accuracy and uploaded into the software, where they were analyzed for common themes according to the content of the interview data.

## Results

A total of 6 rounds of interviews were conducted, consisting of 18 total participants. Each round of interviews consisted of one patient, one intern, and one clinician. Table 1 below shows a breakdown of demographic/background information of participants.

**Table 1:** Participant demographics

	Patients	Interns	Clinicians
Age	32.7	24.5	38.7
Gender (M/F)	3/3	4/2	2/4
Treatment Visit #	2.5	N/a	N/a
Previous Chiropractic Experience (Y/N)	4/2	N/a	N/a
Years of Practice Experience	N/a	N/a	12.7

The most common reason for patients seeking care was for non-specific low back pain (4/6). One patient was being seen primarily for neck pain and another for mid back pain. The majority of patients had previous experience with chiropractic treatment in the past. All patients were treated with HVLA SMT to the region of their primary complaint.

After analyzing the interview data, the following themes were identified: Treatment Outcome, Therapeutic Alliance, Adjunctive Therapies, Significance of Cavitation, and Psychomotor Skills. The themes and their corresponding subthemes are outlined in Table 2 and have been further described below.

**Table 2:** Themes and sub-themes/indication of responses

	Patient	Intern	Clinician
**Treatment outcome**			
Immediate Perceived Improvement	X	X	X
Objective Measures of Change		X	X
**Therapeutic alliance**			
Confidence of Interns			X
Communication Skills	X	X	X
**Adjunctive therapies**			
Complementary Passive Procedures	X	X	
Active Care Plans	X	X	
**Significance of cavitation**			
Indication of Success	X	X	X
Indifference toward Cavitation	X	X	X
Cognitive Dissonance	X	X	
**Psychomotor skills**			
Patient Positioning			X
Appropriate Preload			X
Thrust Specifications			X

### Treatment outcome

When asked about their overall impression of how they perceived the treatment and whether or not that treatment was successful, many participants seemed to be primarily concerned with the immediate outcome following the treatment. Outcomes were divided into subjective perception of improvement and objective measures of change. Patients often emphasized the importance of pain relief immediately following their treatment. Interns also valued the patients’ perceived level of improvement in symptoms, and additionally often utilized objective measures (joint mobility, ranges of motion, orthopedic testing) to assess the effectiveness of the SMT that was delivered.

### Subjective perception of improvement

“I left with less pain than I came in with… I definitely value coming in with stiffness and pain and leaving with less.” - Patient 1.

“It’s essentially about how I feel afterwards, after the overall visit- which is always good.” – Patient 4.

### Objective measures of change

“I always try to do some type of before and after measure- either range of motion, orthopedic testing, tenderness to palpation afterwards… to make sure that what I’m doing is effective, and also for the patient to get a… quantitative measure.” – Intern 4.

“After I adjusted her I re-checked the ranges of motion in the thoracic spine and she felt better, and the range of the motion improved.” - Intern 6.

### Therapeutic alliance

Participants from all three groups seemed to appreciate the importance of developing a therapeutic alliance between patient and provider. The most common component of the therapeutic alliance that was mentioned pertained to the interns’ communication skills. Clinicians also remarked on the importance of the level of confidence portrayed by the intern during patient encounters. Patients often mentioned that they valued well-developed communication skills from their intern. In particular, patients appreciated when the intern communicated a clear explanation of the diagnosis, prognosis, treatment procedures, and timeline for recovery.

### Confidence of interns

“The intern had a very nice control in the room, and the patient was really listening to him… he’s got a confidence about him that some of the interns don’t seem to have.” – Clinician 2.

“I would note that the intern seemed to be nervous, with some shaky hands, during [the adjustment]… which might have affected the success of today’s treatment.” - Clinician 3.

### Communication skills

“I really valued her listening to me and understanding what the question was that I’m asking and being able to answer satisfactorily.” – Patient 4.

“I liked that [the intern] asked me about my pain and was very clear when talking through what she was doing… she explained what she was going to do and what the effects might be later.” - Patient 6.

### Adjunctive therapies

Participants from each group, particularly the patient group, emphasized the importance of receiving adjunctive therapies in addition to SMT. These therapies were subdivided into complementary passive procedures and active care/home exercise plans. Several patients enjoyed the addition of passive therapies such as soft tissue treatment to complement the SMT procedure. Participants also seemed to appreciate the addition of therapeutic exercise as a means to aid in recovery and provide patients with self-management strategies they can perform on their own.

### Complementary passive procedures

“I’ve always needed massage to kind of loosen things up… so I’m very familiar with it, and was excited that [the intern] brought that up. We saw… that it would be beneficial. I know it is beneficial.” – Patient 4.

“I decided to cup before I adjusted it to help loosen up those muscles, and I think that helped the adjustment go more smoothly because it was more loosened up.” - Intern 6.

### Active care/home exercise plans

“I definitely valued the stretches that I got to take home the most, because I can only come here pretty much twice a week, and those adjustments really help, but like I said, I’m only here twice a week… that way if I do feel pain at home, it’s a little remedy for me.” - Patient 1.

“I think giving the patient something that they can do at home to relieve some of the pain that they might be having was really helpful… giving them something that they can take home and use whenever they want I feel like is empowering for their own healthcare autonomy.” - Intern 4.

### Significance of cavitation

Interestingly, there seemed to be mixed responses as to whether or not the presence of cavitation was related to the effectiveness of the adjustment. Some participants valued the audible cavitation as an indicator of a successful maneuver. Others seemed indifferent to the cavitation, and placed greater emphasis on other factors, such as patient comfort during the maneuver and outcome following the treatment.

While clinicians expressed more firm beliefs as to whether or not the presence of cavitation was an important factor, patients and interns were engaged in cognitive dissonance at times. Participants from both the patient and intern groups mentioned conflicting thoughts on the significance of cavitation. For example, despite being told that an audible pop is not likely related to the therapeutic effect associated with SMT, several patients expressed that they still prefer hearing one. Similarly, some interns stated that although they know cavitation might not contribute to the treatment outcome, they appreciate the feedback and confidence that the audible pop provides.

### Audible cavitation as indication of success

“[The adjustment] didn’t feel bad. It felt like it was stretching it at least a little bit. It wasn’t quite as satisfying as when you do get the pop, but I understand it doesn’t always happen.” - Patient 3.

“I certainly liked to hear the cavitation. I value that more… just given the patient’s age and mobility. If they were an older, more arthritic individual I’d be less concerned with an audible cavitation.” – Clinician 1.

### Indifference toward cavitation

“I didn’t feel any popping, but definitely a good stretch… I didn’t really care about hearing a pop.” – Patient 1.

“To me, [the cavitation] was not important, given the nature of what the cavitation is.” - Clinician 3.

### Cognitive dissonance

“I like to hear a pop, but I know it shouldn’t matter. I do find it satisfying… but I know I shouldn’t care.” - Patient 6.

“It’s that instant gratification on my end that I was able to get something to move… I’m trying to recondition myself that… hearing it is not always important. Yes, it’s good. It’s good because the patient can hear it. They know that something moved.” – Intern 3.

### Psychomotor skills

A primary concern of clinicians tasked with observing SMT procedures addressed the psychomotor skills related to the maneuver. These specific skills fell into one of three major categories: patient positioning, appropriate preload force/depth, and specifications of the thrust maneuver itself. Thrust specifications mentioned by clinicians included the amount of force, speed, and line of drive implemented to deliver the thrust.

### Patient positioning

“I certainly considered patient position as one of the factors I looked at when assessing the student’s adjustment.” - Clinician 3.

“The attention to patient positioning through the intern was the secondary thing of value that I noticed… For example, during the prone thoracic, making sure that the leg piece was up at the proper level to reduce that tension through the musculature in the back.” - Clinician 4.

### Appropriate preload

“I’ve been looking at how my interns set up their adjustment, and then specifically how they preload, or take out joint motion before they thrust. In this case I thought the intern did a great job.” – Clinician 5.

“I thought [the intern] took appropriate tissue slack in that area… so I felt like that helped make it easier to adjust that segment.” - Clinician 6.

### Thrust specifications

“I looked at the technique the intern was using… I am kind of calculating line of drive and force used while I observe.” – Clinician 2.

“The patient seemed happy and satisfied with the adjustment. There was a successful drop- he didn’t have to set up multiple times… so I felt like it was a smooth treatment.” - Clinician 3.

## Discussion

This qualitative study identified five major themes related to the factors that contribute to the perceived effect of treatments involving the use of SMT in patients with spine pain. In addition to the specific psychomotor skills required to deliver an effective manipulation, patients, interns, and clinicians highlighted the importance of other factors involved in the patient encounter that can potentially contribute to the perceived effect of the treatment. These factors included developing a strong therapeutic alliance between the patient and intern and using adjunctive therapies in conjunction with SMT. All parties involved seemed to emphasize the importance of immediate subjective and/or objective improvements following the session as an indication of a successful treatment. Treating interns frequently mentioned functional changes following treatment and noted visual or palpable changes from their perspective. The significance of a cavitation during the maneuver was equivocal; some participants seemed to value the presence of cavitation, while others did not.

This study’s findings are consistent with others that have explored patients’ perceptions and values during treatment sessions that include SMT. Similar to the findings from Maiers et al. [14], communication and the therapeutic relationship between patient and provider seemed to play an important role in the perceived success of the treatment session. Many participants also valued the communication skills of the provider, particularly when explaining the diagnosis, prognosis, and treatment procedures. This is consistent with findings derived from semi-structured interviews conducted and analyzed by Plank et al. [15].

Clinicians observing SMT procedures included in this study often mentioned the skills associated as important factors they considered when determining the apparent success of the maneuver. Many of the specifications mentioned were consistent with conclusions from a consensus Delphi study conducted by O’Donnell et al. [28]. These observed factors included components such as patient position and comfort before and during the maneuver, appropriate hand contact and body position displayed by the intern, and generation of the force and speed necessary to successfully deliver the thrust.

Participants of this study viewed the cavitation associated with SMT in different ways. Some patients, interns, and clinicians valued the cavitation as an indicator of a successful maneuver, while others were indifferent and placed value on other themes. Some participants from the patient and intern groups expressed mixed feelings about the cavitation, indicating that while they know cavitation may not play a significant role in the treatment outcome, they still prefer to hear the sound. While the presence of cavitation does not seem to contribute to pain outcomes [29], the possibility exists that expectations and previous experiences play an important role in whether or not the cavitation is viewed as important.

These findings have implications for both clinical and educational practices associated with the use and teaching of SMT. Educational practices should adapt to the modern understanding of SMT as it continues to evolve. A recently-proposed framework highlights the need for modernization of manual therapy teaching practices, and can be used to create a more patient-centered understanding of the delivery of manual therapy techniques [19]. In addition to the physical delivery of SMT, educators should emphasize the importance of the context in which SMT is delivered. Students learning how to perform this skill should be taught to develop a strong therapeutic alliance with patients as they incorporate manual therapy into a treatment plan. Skills related to the development of communication and confidence are paramount for trainees when developing a working alliance with patients in a clinical setting [30]. Therefore, educators should work to develop these skills in parallel with the psychomotor skills necessary to perform SMT.

From a clinical standpoint, the occurrence and distribution of themes and subthemes among participants demonstrates that patients and doctors often hold differing beliefs about treatment and may value different components of the therapeutic encounter. This is important for clinicians to acknowledge, as recognizing this reality could potentially open communication channels and help to identify patients’ values and expectations that might bolster the patient’s experience and their perceived treatment effect. These findings also highlight the importance of contextual factors surrounding the delivery of SMT. Chiropractors and other manual therapists who use SMT in practice should take advantage of the other factors that might play a role in the overall perceived treatment effect, such as the therapeutic alliance with patients, which is known to contribute to positive outcomes in patients with spine pain [31, 32]. Additionally, SMT should be used in conjunction with other adjunctive therapies, both active and passive, to align with patients’ expectations and involve them as active participants in their care plans.

There are several limitations in this study to be addressed. First, the participants enrolled came from a single chiropractic teaching clinic which may limit the transferability of findings, or the degree to which findings can be applied in another context or setting [33]. The semi-structured interviews were also conducted by a single researcher, who analyzed the data and developed the themes and subthemes described in the results. Another limitation is the prior relationships which existed between the researcher and clinician/intern participants, as the researcher was also a faculty member at the institution at which the study was conducted. This may have influenced participant recruitment as well as their responses during the interview process. No participants who were invited to participate in the study declined, which is likely attributable to the relationship of the researcher as clinician, teacher, and colleague to the participants.

Future research should investigate the extent to which current SMT teaching practices incorporate the role of other factors in the treatment effect associated with SMT. The relationship between the themes identified and patients’ responses to treatment involving the use of SMT is another area that could be explored. Individual patients, students, and practitioners seem to uniquely emphasize some areas associated with treatment effectiveness over others. Investigating the relationship between these specific factors and the treatment response further could provide greater insight into patient-specific responses to treatment.

## Conclusion

This qualitative study identified several themes which describe factors that may contribute to the perceived effect associated with SMT. These findings could have implications for the utilization and teaching of the principles of SMT. In addition to the psychomotor skills required to perform SMT, educators and practitioners should consider factors such as the therapeutic alliance between patient and provider, use of adjunctive therapies, and assessment of the outcome associated with the intervention.

## Electronic Supplementary Material

Below is the link to the electronic supplementary material.

Supplementary Material 1

## Data Availability

The datasets generated and/or analysed during the current study are not publicly available due to confidentiality of transcript information but are available from the corresponding author on reasonable request.

## Abbreviations

SMT: Spinal Manipulative Therapy
HVLA: High-velocity low-amplitude
