# Supplementary material for: Factors that contribute to the perceived treatment effect of spinal manipulative therapy in a chiropractic teaching clinic: a qualitative study
Source: Chiropr Man Therap. 2024 Dec 18;32:41. doi: 10.1186/s12998-024-00554-z (PMC11658066; doi:10.1186/s12998-024-00554-z)
Supplement: Supplementary file 1 — Supplementary Material 1 [file 12998_2024_554_MOESM1_ESM.pdf]

Supplementary File 1: Interview Guides

Patient Version

- How do you feel the treatment, particularly the manipulation or adjustment, went today?
- What specific factors did you consider when deciding your answer?
- How were the treatment and its effects explained to you the by intern or clinician?
- Did you hear or feel a “pop” during the manipulation/adjustment?
- If yes, how was this explained to you by the intern and/or clinician?
- Was hearing or feeling the “pop” important to you?
- Were there any aspects of the treatment that you valued the most?
- Overall, would you consider the treatment rendered today "successful”?
- What do you think played a role in your decision?

Intern/Clinician Version

- How do you feel the treatment, particularly the manipulation or adjustment, went today?
- What specific factors did you consider when deciding your answer?
- How were the treatment and its effects explained to the patient?
- Did you hear or feel a “pop” during the manipulation/adjustment?
- If yes, how was this explained to the patient?
- Was hearing or feeling the “pop” important to you?
- Were there any aspects of the treatment that you valued the most?
- Overall, would you consider the treatment rendered today "successful”?
- What do you think played a role in your decision?
